# Folate receptor alpha in ovarian cancer tissue and patient serum is associated with disease burden and treatment outcomes

**DOI:** 10.1038/s41416-022-02031-x

**Published:** 2022-11-19

**Authors:** Heather J. Bax, Jitesh Chauhan, Chara Stavraka, Aida Santaolalla, Gabriel Osborn, Atousa Khiabany, Melanie Grandits, Jacobo López-Abente, Lais C. G. F. Palhares, Charleen Chan Wah Hak, Alexandra Robinson, Amy Pope, Natalie Woodman, Cristina Naceur-Lombardelli, Sadek Malas, Jack E. M. Coumbe, Mano Nakamura, Roman Laddach, Silvia Mele, Silvia Crescioli, Anna M. Black, Sara Lombardi, Silvana Canevari, Mariangela Figini, Ahmad Sayasneh, Sophia Tsoka, Kevin FitzGerald, Cheryl Gillett, Sarah Pinder, Mieke Van Hemelrijck, Rebecca Kristeleit, Sharmistha Ghosh, Ana Montes, James Spicer, Sophia N. Karagiannis, Debra H. Josephs

**Affiliations:** 1 St. John’s Institute of Dermatology, School of Basic & Medical Biosciences, King’s College London, Guy’s Hospital, London, UK; 2 School of Cancer & Pharmaceutical Sciences, King’s College London, Guy’s Hospital, London, UK; 3 Cancer Centre at Guy’s, Guy’s and St Thomas’ NHS Foundation Trust, London, UK; 4 Translational Oncology & Urology Research (TOUR), School of Cancer & Pharmaceutical Sciences, King’s College London, Guy’s Hospital, London, UK; 5 King’s Health Partners Cancer Biobank, School of Cancer & Pharmaceutical Sciences, King’s College London, Guy’s Hospital, London, UK; 6 Department of Informatics, Faculty of Natural, Mathematical & Engineering Sciences, King’s College London, Bush House, London, UK; 7 Guy’s and St Thomas’ Oncology & Haematology Clinical Trials (OHCT), Guy’s Cancer Centre, Guy’s and St Thomas’ NHS Foundation Trust, London, UK; 8 Fondazione IRCCS Istituto Nazionale dei Tumori Milano, Milan, Italy; 9 Biomarker Unit, Dipartimento di Ricerca Applicata e Sviluppo Tecnologico (DRAST), Fondazione IRCCS Istituto Nazionale dei Tumori, Milan, Italy; 10 Epsilogen Ltd., Waterfront, ARC West London, Manbre Road, Hammersmith, London, UK; 11 Breast Cancer Now Research Unit, School of Cancer & Pharmaceutical Sciences, King’s College London, Guy’s Cancer Centre, London, UK

**Keywords:** Ovarian cancer, Tumour biomarkers

## Abstract

**Background:**

Survival rates for ovarian cancer remain poor, and monitoring and prediction of therapeutic response may benefit from additional markers. Ovarian cancers frequently overexpress Folate Receptor alpha (FRα) and the soluble receptor (sFRα) is measurable in blood. Here we investigated sFRα as a potential biomarker.

**Methods:**

We evaluated sFRα longitudinally, before and during neo-adjuvant, adjuvant and palliative therapies, and tumour FRα expression status by immunohistrochemistry. The impact of free FRα on the efficacy of anti-FRα treatments was evaluated by an antibody-dependent cellular cytotoxicity assay.

**Results:**

Membrane and/or cytoplasmic FRα staining were observed in 52.7% tumours from 316 ovarian cancer patients with diverse histotypes. Circulating sFRα levels were significantly higher in patients, compared to healthy volunteers, specifically in patients sampled prior to neoadjuvant and palliative treatments. sFRα was associated with FRα cell membrane expression in the tumour. sFRα levels decreased alongside concurrent tumour burden in patients receiving standard therapies. High concentrations of sFRα partly reduced anti-FRα antibody tumour cell killing, an effect overcome by increased antibody doses.

**Conclusions:**

sFRα may present a non-invasive marker for tumour FRα expression, with the potential for monitoring patient response to treatment. Larger, prospective studies should evaluate FRα for assessing disease burden and response to systemic treatments.

## Background

Ovarian cancer is the third most common but most lethal gynaecological malignancy worldwide. Non-specific symptomatology and lack of validated screening tools often result in late-stage diagnosis [1]. Whilst a combination of surgical debulking and platinum/taxane-based chemotherapy followed by maintenance PARP inhibitors and/or bevacizumab [2–5] is the mainstay of treatment for advanced disease, most women subsequently relapse, resulting in 5-year survival rates of 13–27% for patients with late-stage (III–IV) disease [6].

Currently, CA125 is the most widely used serum biomarker for monitoring ovarian cancer. However, intra‐tumour heterogeneity, a key feature of ovarian tumours [7], suggests the presence of distinct sub-clonal populations within a tumour, possibly harbouring different marker phenotypes. Therefore, incorporating several complementary biomarkers that may account for the molecular heterogeneity of ovarian cancer sub-clones as well as different histotypes could improve surveillance and evaluating response to treatment [8].

FRα is a glycosylphosphatidylinositol (GPI)-anchored cell-surface glycoprotein encoded by the *FOLR1* gene. FRα binds with high affinity to folic acid and its derivatives [9] and mediates cellular processes, including cell division, proliferation, and tissue growth via signalling cascades and components of the folate cycle [10–12]. The distribution of FRα across non-malignant tissues is limited; however, elevated expression has been observed in several cancers, including ovarian, endometrial, lung and breast cancer subsets [13–19]. Up to 90% of ovarian cancers overexpress FRα [20, 21]; however, differential levels of expression have been observed across different ovarian cancer histotypes [22]. FRα can be shed from tumour cells and is detected in the circulation in soluble form (sFRα) [23–25]. Given that FRα is highly expressed in ovarian cancer, and is readily measurable in the serum, sFRα may have the potential to be leveraged as an adjunct clinical biomarker for the disease.

In addition to its potential role as a clinical biomarker, there has been significant interest in using FRα as a therapeutic target in patients with ovarian cancer, with several FRα-targeted therapeutics, such as farletuzumab and mirvetuximab soravtansine, in late-phase trials [26–31]. Despite promising results, FRα-targeted agents are yet to be approved for use in patients with cancer. This may in part be attributed to the challenge of selecting patients who are likely to respond. Immunohistochemical staining of archival tumour samples is commonly used to determine FRα protein expression levels for the inclusion of patients in clinical trials [32]. Additionally, sFRα-based assessment of FRα tumour expression might complement the selection of patients for FRα-targeted therapeutics. Presently, understanding the correlations of circulating levels of sFRα with tumour volume and/or the degree of tumoural FRα expression, and whether sFRα levels are influenced by treatments, are still required before this approach can be implemented clinically.

In this study, we performed immunohistochemical staining for FRα expression in tumours to investigate the relationship between sFRα and tumour FRα status and to evaluate the possible application of sFRα testing in future clinical studies of FRα-targeted therapies. We investigated sFRα levels in ovarian cancer patients longitudinally, before and during treatment with neo-adjuvant, adjuvant and palliative systemic therapies, as well as in relation to concurrent tumour burden. Tumour burden was defined by a bespoke scoring system designed for this study, with the aim of capturing the volume of disease in its entirety. Furthermore, we asked whether free FRα could impact the efficacy of anti-FRα treatments.

## Methods

### Analysis of *FOLR1* expression in normal tissues and ovarian cancer

Gene expression of *FOLR1* (encoding FRα) by various cancer cell lines, tumours and normal tissues were interrogated using the Cancer Cell Line Encyclopedia (CCLE) online tool (www.broadinstitute.org/ccle), The Pathology Atlas of The Human Protein Atlas online tool [33, 34] (https://www.proteinatlas.org/humanproteome/pathology), and Xenabrowser online tool [35] (Xenabrowser.net), respectively. Gene expression of *FOLR1* and molecules in the folate/FRα signalling pathway: MTHFR, methylene tetrahydrofolate reductase; FOLH1, glutamate carboxypeptidase; TYMS, thymidylate synthase; DHFR, dihydrofolate reductase, were studied in normal ovary and ovarian cancer tissues using the Gene Expression Profiling Interactive Analysis (GEPIA) online tool [36] (http://gepia.cancer-pku.cn/index.html).

### Sample collection pathway to evaluate FRα in tumour tissues and patient blood

Sample collection for this study was reviewed and approved by the Guy’s Research Ethics Committee (Reference 09/H0804/45 and 16/LO/0366) and performed at Guy’s Hospital in London UK. Ovarian cancer patients and healthy volunteers were enrolled by written informed consent. Ovarian cancer patients who had undergone screening for potential inclusion in an anti-FRα clinical trial provided archival tumour tissue (*N* = 316); of these, *N* = 55 also provided blood samples. An additional cohort of *N* = 88 patients was approached during outpatient clinic appointments and provided blood samples only.

**Fig. 1:**
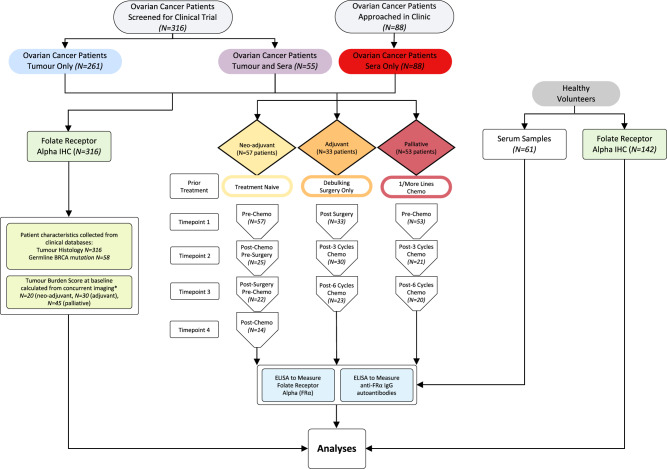
Study design and sample workflow. Serum samples from ovarian cancer patients were collected at timepoint 1 and up to 3 sequential treatment-related time points (e.g., for the treatment-naïve group: pre-chemo, post-chemo/pre-surgery, post-surgery/pre-chemo, and post-chemo). These were collected alongside serum samples from healthy volunteers. Serum samples were studied for sFRα and anti-FRα autoantibodies, and FRα protein expression in patient tumours and normal tissue microarrays was evaluated by immunohistochemistry. Patient characteristics, including tumour histotype, were collected from clinical databases (see Supplementary Table 1 for ovarian patients in the longitudinal study). Patient tumour burden scores were calculated from concurrent imaging (*see Supplementary Table 2 for a scoring system). *N* numbers are indicated in the figure.

To study tumour-expressed and sFRα in ovarian cancer patients, we established a protocol to collect and analyse tumour tissue (*N* = 316 patients) and serum (*N* = 143 patients) samples (Fig. 1). Where possible, longitudinal serum samples were collected at defined timepoints throughout standard treatment regimens. Furthermore, these were evaluated with reference to patient characteristics from clinical databases; namely tumour histotype, germline BRCA1/2 mutation status (Supplementary Table 1), and tumour burden score (calculated from concurrent imaging as outlined in Supplementary Table 2). Sera from healthy volunteers (*N* = 61) were also collected and studied as controls.

Serum samples were prepared by centrifugation of clotted blood in SST Clot Activator and Polymer Gel Hemogard Closure Blood Tubes (BD) at 2500 RPM for 15 min at 4 °C and stored at 80 °C until analysis.

### Tumour expression of FRα by immunohistochemistry (IHC)

Immunohistochemistry (IHC) was used to evaluate the membrane and cytoplasmic expression of FRα by formalin-fixed, paraffin-embedded normal tissue sections in tissue microarrays and tumour sample sections from ovarian cancer patients [15, 37]. Novocastra^TM^ Liquid mouse anti-human FRα primary antibody (clone BN3.2, Leica) was applied to formalin-fixed, paraffin-embedded tumour sections (from primary debulking surgery) for 32 min at room temperature at 1/500 dilution, followed by detection with Ultra Universal 3,3’-diaminobenzidine (DAB) detection kit (Ventana Medical Systems Inc.) and then Haematoxlyin II applied for 8 min. Scoring of tissue sections was performed by pathologists. FRα positivity was determined as ≥5% staining in the membrane and/or cytoplasm (at any intensity). Scoring is also presented as staining on the membrane of 0–<5, 5–<25, 25–<50 and ≥50% tumour cells (at any intensity), and as *H* scores (calculated by multiplying the % of cells with membrane expression with the intensity of staining scored 0–3; maximum *H* score of 300) [38].

### Serum FRα and anti-FRα autoantibodies

ELISAs were performed to evaluate the concentrations of circulating sFRα and anti-FRα IgG autoantibodies in serum samples from ovarian cancer patients and healthy volunteers [37, 39]. MaxiSORP™ plates (Nunc) were coated with 100 μl/well of 2 μg/ml monoclonal mouse anti-human FRα IgG1 antibody (clone 548908) or 1 μg/ml recombinant FRα, respectively (both R&D Systems). Following incubation at 4 °C overnight, 250 μl/well SuperBlock^TM^ (Perbio Science Ltd.) was added for 2 h at room temperature (RT). Serum samples were diluted to 20% in a 50:50 solution of SuperBlock^TM^ and PBS-0.05% Tween® 20 solution (Severn Biotech and Sigma, respectively). Standard curves of recombinant FRα (R&D Systems), or anti-FRα human IgG1 monoclonal antibody (prepared in house) were diluted in SuperBlock^TM^-PBS-0.05% Tween® 20, supplemented with 20% human serum albumin (type AB male, Sigma). Samples and standards were added 50 μl/well, in triplicate, and incubated for 2 h at RT. FRα was detected by 50 μl/well biotinylated polyclonal goat anti-human FRα IgG1 antibody (R&D Systems, diluted to 25 ng/ml in SuperBlock^TM^-PBS-0.05% Tween® 20) for 2 h at RT, followed by 50 μl/well streptavidin-peroxidase conjugate (Pierce, diluted 1/22,000 in SuperBlock^TM^-PBS-0.05% Tween® 20) for 30 min at RT. Anti-FRα IgG autoantibodies were detected by 50 μl/well HRP-conjugated polyclonal goat anti-human Fcγ-specific F(ab’)_2_ fragment (Jackson Immuno Research, diluted 1/500 in SuperBlock^TM^-PBS-0.05% Tween® 20) for 45 min at RT. Plates were developed with 50 μl/well OPD (Sigma) diluted to 0.5 mg/ml in stable peroxidase substrate buffer (Pierce), for 5–10 min at RT, in darkness, followed by 50 μl/well 1 M HCl solution (Sigma). Washing at each step was performed with 250 μl/well PBS-0.05% Tween® 20 4 times. A Fluostar Omega microplate reader (BMG LABTECH) was used with an absorbance of 492 nm, and correction wavelength of 650 nm. Standard curves were fitted using a 4-point variable curve-fitting program using a minimum of 6 points (MARS software, BMG LABTECH). The lower levels of quantification (LLOQ) were 6.25 and 3.125 ng/ml, respectively. Values below LLOQ are reported as 0 ng/ml. Each sample was analysed in technical triplicates and data are reported as the mean of two independent experiments.

### Tumour burden score

Tumour burden scores were calculated from concurrent imaging as outlined in Supplementary Table 2. Briefly, total scores were determined by the sum of points calculated according to the number of sites of disease, the number of metastases per site, and the maximum metastasis diameter per site.

### In vitro efficacy of FRα-targeting therapy

Cell-surface binding of an FRα-specific IgE therapeutic candidate, MOv18 IgE, to FRα-expressing IGROV1 ovarian cancer cells (CVCL_1304, Sigma) was confirmed by incubation of 1 × 10^5^ cells with indicated concentrations of MOv18 IgE for 30 min at 4 °C, followed by FITC-conjugated polyclonal goat anti-human IgE antibody (1/50 dilution; Vector Laboratories) for 20 min at 4 °C. An established flow cytometric antibody-dependent cellular cytotoxicity (ADCC) and phagocytosis (ADCP) assay was used, as previously described [40, 41], to evaluate the efficacy of a FRα-specific IgE therapeutic candidate, MOv18 IgE, in the presence of soluble FRα. IGROV1 cancer cells were incubated with human peripheral blood mononuclear cells (PBMCs) or U937 human monocytic cells (CRL-1593.2, ATCC) (at ratios of 1:10 and 1:1.5, respectively), together with indicated concentrations of MOv18 IgE (in-house) and recombinant FRα antigen (R&D Systems). Cell lines were routinely tested for mycoplasma contamination by PCR. Flow cytometric acquisitions were performed on a FACS Canto II using FACSDiva software, and analyses were conducted using the FlowJo software (FlowJo LLC, BD). Each condition was analysed in technical triplicates and data are reported as the mean of four independent experiments.

### Statistical analyses

For in vitro efficacy assays, with equal sample size, and assuming 90% power and a significance level of 5%, we calculated that at least 3 independent experiments are required for each group to detect a difference between conditions. For analyses of patient serum and tumour tissues, a minimum of 5 samples were compared (90% power and a significance level of 5%). No samples were excluded from the analysis. Descriptive statistical analyses of the data were performed to calculate EC50, as well as group comparisons using non-parametric Mann–Whitney *T* test, one-way analysis of variance with non-parametric Kruskal–Wallis multiple comparisons and Chi-square test. Beta coefficient estimates for change in sFRα and tumour burden score between longitudinal timepoints were calculated using linear regression. Linear regression analyses were also performed to assess whether the variation in sFRα over the treatment course, was associated with the variation of the tumour burden score. Receiver operating characteristic (ROC) curve analyses were performed to assess the prediction capabilities of: (i) sFRα for tumour expression of FRα, and (ii) sFRα for high tumour burden score. The correlation between concurrent tumour burden score and sFRα levels was assessed by Pearson’s correlation coefficient. All the analyses were conducted using GraphPad Prism 9 or IBM SPSS statistics 27. Test significance (*P* value) is represented as follows: *P < 0.05, **P < 0.01, ***P < 0.001, ****P < 0.0001. Error bars represent the standard error of mean (SEM).

### Reporting summary

Further information on research design is available in the Nature Research Reporting Summary linked to this article.

## Results

### High FRα gene expression in ovarian cancer compared with other tumour types and normal tissues

**Fig. 2:**
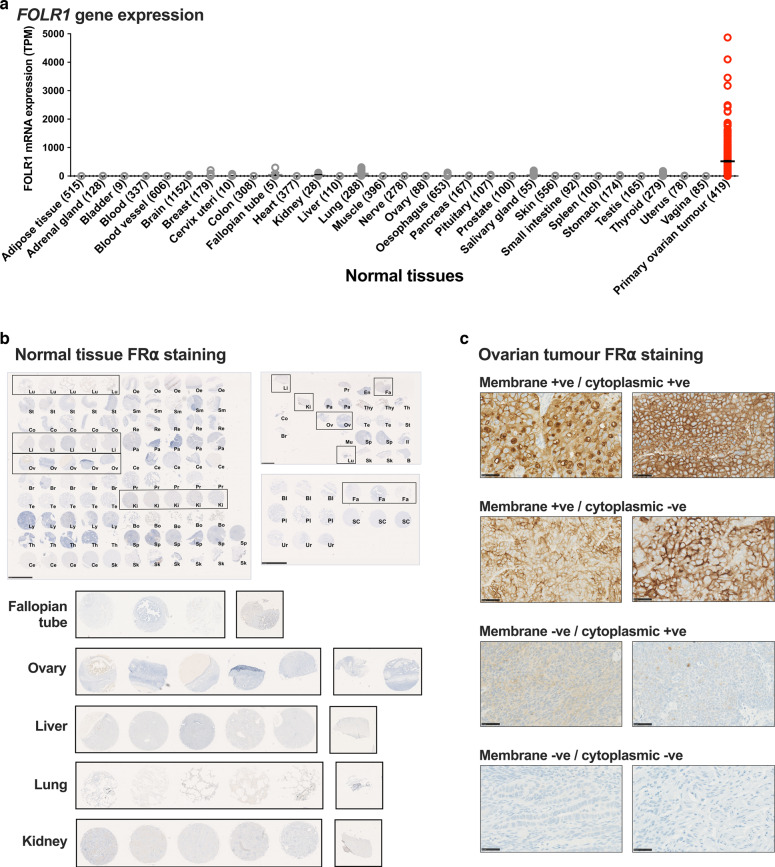
Transcriptomic and immunohistochemical analyses of FRα expression in normal tissues and ovarian tumours. **a** Low *FOLR1* gene expression in normal tissues (grey) compared to primary ovarian tumours (red) (N numbers indicated in parentheses; The Human Protein Atlas online tool Xenabrowser online tool [35] (Xenabrowser.net). **b** No membrane or cytoplasmic FRα protein expression on a broad range of normal tissues (*N* = 142; scale bars = 2.5 mm). Key normal tissue types; fallopian tube (Fa), ovary (Ov), liver (Li), lung (Lu) and kidney (Ki) are highlighted with black boxes and zoomed images shown. **c** Representative images of ovarian tumour FRα staining (×40 magnification; scale bar = 50 μm). Bl bladder, Bo bone marrow, B brain, Br breast, Cb cerebrum; Ce cervix; Co colon; En endometrium, Fa fallopian tube, Il ileum, Ki kidney, Li liver, Lu lung, Ly lymph node, Mu muscle, striated, Oe oesophagus, Ov ovary, Pa pancreas, Pl placenta, Pr prostate, Re rectum, Sk skin, Sm small intestine, SC spinal cord, Sp spleen, St stomach, Te testis, Th thymus, Thy thyroid, Ur ureter.

We confirmed the expression of FRα across ovarian cancer cell lines and tissue specimens, several other cancer types, and normal tissues, by interrogating publicly available data [33–35]. *FOLR1* (encoding FRα) was highly expressed in human ovarian cancer cell lines and ovarian cancer specimens, compared to other cell lines and cancers (Supplementary Fig. 1A, B). In line with the known roles of folate/FRα pathway in cell growth, proliferation and survival [11, 12] (Supplementary Fig. 1C), we found dysregulated expression of folate pathway genes in ovarian cancer [36]: gene expression of *FOLR1*, TYMS (thymidylate synthase) DHFR (dihydrofolate reductase) were significantly-greater, and gene expression of MTFHR (methylene tetrahydrofolate reductase) was lower in tumours (*N* = 426), compared to normal ovary (*N* = 88) (Supplementary Fig. 1D). *FOLR1* expression was markedly higher in primary ovarian tumour samples compared to a range of normal tissues [35] (Fig. 2a). These data suggest that *FOLR1* is highly expressed in ovarian and other cancers compared with normal tissue.

### Immunohistochemical analyses reveal a mixture of cell surface and cytoplasmic FRα protein expression in ovarian cancer tissues

**Fig. 3:**
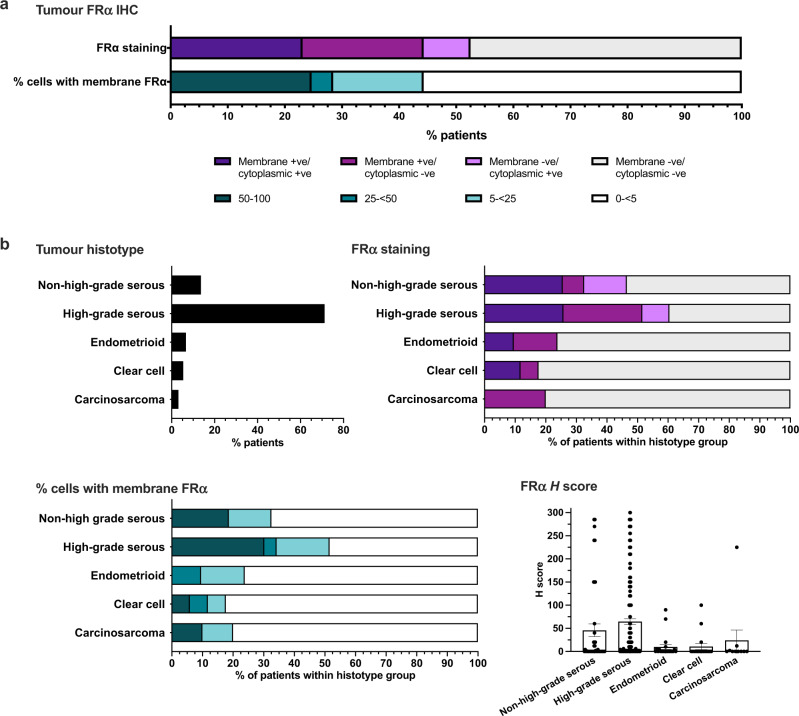
Immunohistochemical analyses reveal a mixture of cell surface and cytoplasmic FRα protein expression in ovarian cancer tissues. **a** Percentage of patients with tumours expressing FRα on the membrane, in the cytoplasm, both or neither, and with membrane staining on 0–<5, 5–<25, 25–<50 or ≥50% of tumour cells. **b** Top left: Overall percentage of patients with each tumour histotype; Top right: Percentage of patients with membrane and cytoplasmic positivity within each histotype subgroup; Bottom left: Percentage of patients within each histotype subgroup with different proportions of membrane positive tumours cells; Bottom right: Immunohistochemical *H* scores calculated in each histotype subgroup.

Having established high levels of *FOLR1* gene expression in ovarian cancer, we evaluated FRα protein expression in normal tissues and ovarian tumours by immunohistochemistry (Figs. 1 and 2; *N* = 142 normal tissues; *N* = 316 ovarian tumours). No membrane or cytoplasmic FRα expression was observed across a range of normal tissues, including normal fallopian tube, ovary, lung, kidney and liver, where FRα expression has been previously reported at luminal surfaces [22, 42] (Fig. 2b). Comparatively, 52.7% of tumours from this diverse patient cohort were positive for either membrane or cytoplasmic staining, or both (Figs. 2c and 3a). Membrane staining of ≥5% tumour cells was observed in 44.2% of patients (5–<25% cells in 15.8% patients, 25–<50% cells in 3.8% patients, and ≥50% cells in 24.6% patients) (Fig. 3a). In our cohort, 71% of tumours were known to have high-grade serous histotype, with other histotypes represented less frequently (Fig. 3b, top left). Sixty percent of the high-grade serous tumours were positive for either membrane or cytoplasmic FRα protein staining in ≥5% of cells (Fig. 3b, top right). In each sample, FRα expression on the membrane was not always associated with expression in the cytoplasm and vice versa, apart from 20% of the tumour samples that demonstrated both membrane and cytoplasmic FRα expression (data not shown). Of the fifty percent of high-grade serous tumours with ≥5% of tumour cells showing membrane FRα expression, 17.3% of tumours showed expression on 5–<25% cells, 4% of tumours on 25–<50% cells and 30.2% of tumours on ≥50% cells (Fig. 3b, bottom left). Furthermore, the immunohistochemical *H* score (calculated from the % of stained cells and staining intensity) was greater for high-grade serous tumours compared to other histotypes (Fig. 3b, bottom right). These findings confirm expression of FRα by ovarian cancers, particularly those with high-grade serous histotype.

### Soluble FRα but not anti-FRα autoantibodies were elevated in the circulation of ovarian cancer patients compared with healthy subjects

**Fig. 4:**
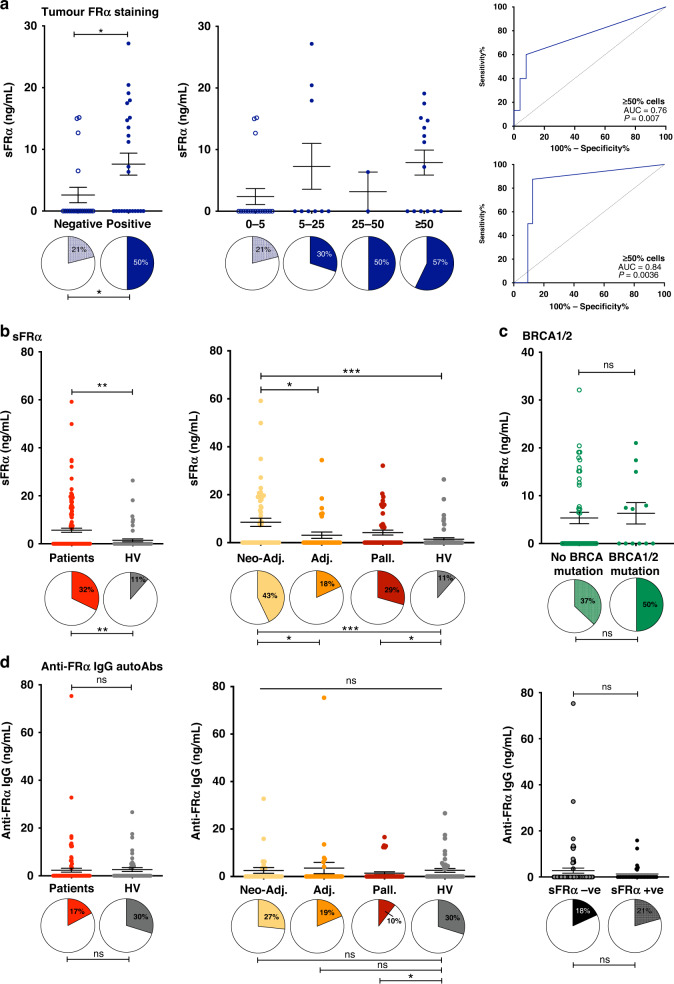
Soluble FRα but not anti-FRα autoantibodies were elevated in the circulation of ovarian cancer patients compared with healthy subjects. sFRα and anti-FRα autoantibodies were measured in three patient cohorts (neo-adjuvant, adjuvant, palliative) at timepoint 1 (see Fig. 1). **a** sFRα levels were significantly-higher in patients with FRα tumour cell membrane expression, compared to patients with FRα-negative tumours (left) and with trend for higher levels in patients with tumours showing FRα cell membrane expression in a greater proportion of tumour cells (middle). In neo-adjuvant treatment-naïve patients, baseline sFRα concentration was predictive of FRα cell membrane expression in both ≥5% and ≥50% of tumour cells (right top and bottom, respectively). **b** Significantly-higher sFRα levels were measured in ovarian cancer patients compared to healthy volunteers, and between patient cohorts (proportion of samples with detectable sFRα indicated by filled pie chart sections below). **c** sFRα levels, or proportions of samples with detectable sFRα, were not associated with the patient’s germline BRCA1/2 mutational status. **d** Comparable levels of anti-FRα autoantibodies were detected in serum samples from ovarian cancer patients and healthy volunteers (left), across all patient cohorts (middle), and irrespective of detectable sFRα (right). *N* numbers in Fig. 1; *P ≤ 0.05, **P ≤ 0.01, ***P ≤ 0.001, ****P ≤ 0.0001. Statistical tests: *t* test, one-way ANOVA with Kruskal–Wallis multiple comparisons, receiver operating characteristic (ROC) curve analyses and Chi square test. Error bars represent the standard error of mean (SEM).

We next measured sFRα and anti-FRα IgG autoantibodies in the circulation of ovarian cancer patients at timepoint 1 (prior to the start of the treatment regimen, Fig. 1), compared with healthy subjects (Fig. 4). In patients where IHC analysis showed tumour cell positivity for FRα (≥5% tumour cells in the sample), the concentration of sFRα and proportion of samples with detectable levels, were significantly greater (7.6 ± 1.8 ng/ml; detectable in 50%), compared to patients with FRα-negative tumours (2.6 ± 1.2 ng/ml; detectable in 21%) (Fig. 4a, left). Furthermore, the proportion of samples with detectable sFRα was greatest in patients with tumours where ≥50% cells expressed FRα on the membrane, and a trend for higher concentration of sFRα was observed in this group, compared to patients with 0–<25% positive tumour cells in tumour lesions (Fig. 4a, middle). Although there was no correlation between sFRα levels and % tumour cells expressing FRα or the IHC *H* score (data not shown), receiver operating characteristic (ROC) curve analysis revealed that the baseline level of circulating sFRα in neo-adjuvant treatment-naive patients was predictive of positive tumour cell membrane expression of FRα (AUC = 0.76, *P* = 0.007 for predicting tumours with ≥5% positive cells and AUC = 0.84, *P* = 0.0036 for predicting tumours with ≥50% positive cells; Fig. 4a, right, top and bottom, respectively). On the other hand, sFRα concentration was not predictive of cytoplasmic FRα expression in the patient’s tumour (AUC = 0.71, *P* = 0.21; Supplementary Fig. 2).

We found significantly-higher concentrations of sFRα in patient (5.7 ± 0.9 ng/ml (mean ± SEM); *N* = 143), compared with healthy volunteer sera (1.4 ± 0.6 ng/ml; *N* = 61), and a higher proportion of samples with detectable levels of sFRα in patients compared to healthy individuals (32 and 11%, respectively) (Fig. 4b, left). sFRα levels, and the proportion of samples with detectable sFRα, were significantly raised at the start of neo-adjuvant chemotherapy (8.5 ± 1.7 ng/ml; detectable in 43%) compared to prior to adjuvant chemotherapy, where patients had recently undergone debulking surgery (3.1 ± 1.3 ng/ml; detectable in 18%) (Fig. 4b, right). At the start of palliative treatment, sFRα also showed signs of elevation (4.2 ± 1.1 ng/ml; detectable in 29%). Furthermore, regardless of the presence of confirmed germline BRCA mutations, sFRα levels were not significantly different, suggesting no clear association with these pathogenic genomic alterations (Fig. 4c).

As elevated levels of sFRα were detected in ovarian cancer, we considered whether patients mounted a humoural response to FRα-expressing tumours and/or circulating sFRα. Anti-FRα IgG autoantibodies were detected in 17% of serum samples from ovarian cancer patients (2.3 ± 0.8 ng/ml), and in 30% from healthy volunteers (2.6 ± 0.8 ng/ml) (Fig. 4d, left). We found no significant differences in anti-FRα autoantibody levels and proportion of samples with detectable anti-FRα autoantibodies across patient cohorts (Fig. 4d, middle), and no differences between patients with or without detectable circulating sFRα (Fig. 4d, right).

### Variations in sFRα are associated with tumour burden

**Fig. 5:**
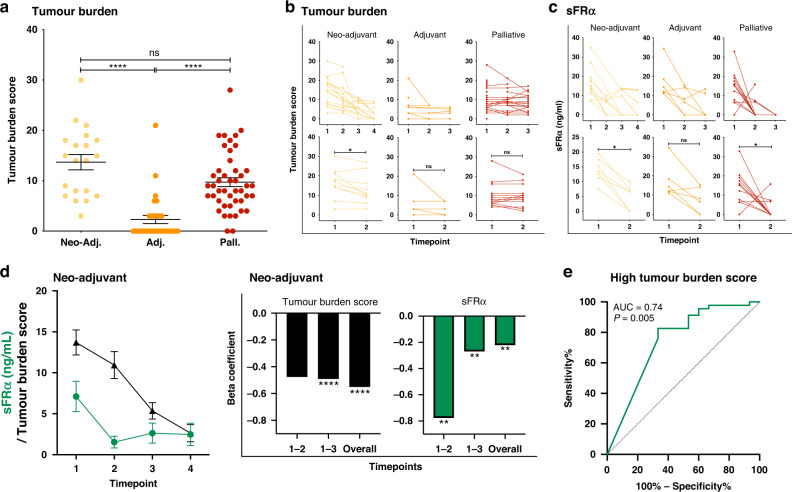
Serum markers and tumour burden score change during patient treatment. **a** Patient tumour burden score at the start of treatment (calculated as described in Supplementary Table 2) was significantly greater in the neo-adjuvant and palliative ovarian cancer patient cohorts, compared to the adjuvant patient cohort. **b**, **c** Changes in tumour burden score (**b**) and levels of sFRα (**c**) were measured across treatment timepoints for all ovarian cancer patient cohorts. **d** (Left) Comparison of changes in serum sFRα and tumour burden score over time in patients receiving standard neo-adjuvant treatment regimen. (Right) Beta coefficients illustrate the variation in sFRα among the different time points, namely timepoints 1 and 2, timepoints 1 and 3, and overall across the earliest and latest timepoint for which data were collected. *N* numbers and treatment timepoints as indicated in Fig. 1. **e** Receiver operating characteristic (ROC) curve analysis evaluating the capacity sFRα to predict high values of tumour burden scores in the neo-adjuvant treatment-naive patient group. sFRα levels were predictive of high tumour burden scores (high defined as the upper quartile: ≥75th percentile of tumour burden scores). *P ≤ 0.05, **P ≤ 0.01, ****P ≤ 0.0001. Statistical tests: *t* test, one-way ANOVA with Kruskal–Wallis multiple comparisons, receiver operating characteristic (ROC) curve analyses and linear regression analysis. Error bars represent the standard error of mean (SEM).

Having confirmed that soluble FRα levels were elevated in the circulation of ovarian cancer patients, we explored whether changes in sFRα levels were indicative of changes in the patients’ disease burden as a response to treatment (Fig. 5). First, we devised a tumour burden scoring system for the purpose of this study, in order to apply a numerical score to the entirety of disease observed by routine clinical imaging at each timepoint in a patient’s treatment regimen (Supplementary Table 2). Tumour burden score was used to capture the volume of disease at all sites, instead of selectively focusing on only the target lesions as captured by RECIST score [43]. Using this scoring system, as expected disease burden was significantly higher in patients at the start of neo-adjuvant and palliative treatment, than in the adjuvant cohort (Fig. 5a). The tumour burden score markedly decreased over the course of neo-adjuvant treatment, with a significant reduction measured between timepoints 1 (treatment naïve) and 2 (post-neo-adjuvant chemotherapy, but before debulking surgery). Although a similar trend was observed over the course of palliative treatment, the change in tumour burden score was not significant (Fig. 5b).

Focussing on patients with detectable levels of circulating sFRα at the start of treatment (timepoint 1), we next evaluated the concentration of sFRα in patient serum samples collected over the course of treatment (timepoints outlined in Fig. 1). Similarly to tumour burden score, sFRα levels decreased over the course of neo-adjuvant and palliative treatment, with significant changes between timepoints 1 and 2, and a downward trend was observed in adjuvant patients (Fig. 5c).

In neo-adjuvant patients, we compared concurrent sFRα and tumour burden scores. For sFRα the greatest decrease occurred between timepoints 1 and 2, whereas the decrease in the tumour burden score appeared to be more gradual over the entire course of treatment (Fig. 5d, left). In order to determine whether a reduction in sFRα was associated with a reduction in tumour burden score, we analysed its change between timepoints over the course of treatment. The beta-coefficient estimates of the linear regression of the marker (Fig. 5d, right) confirmed the pattern of change observed in Fig. 5d left and described above. A modest correlation was observed between tumour burden score and the level of circulating of sFRα (Supplementary Fig. 3; *R*^2^ = 0.14, *P* = 0.004). Receiver operating characteristic (ROC) curve analysis showed that levels of circulating sFRα in neo-adjuvant treatment-naïve patients were associated with high values of tumour burden score (upper quartile: ≥75th percentile) (AUC = 0.74, *P* = 0.005) (Fig. 5e).

These data suggest that longitudinal sFRα levels may be reflective of the patients’ disease burden and changes in disease burden following standard therapies.

### Soluble FRα may partly reduce the anti-tumour functions of a FRα-targeted therapeutic antibody candidate

**Fig. 6:**
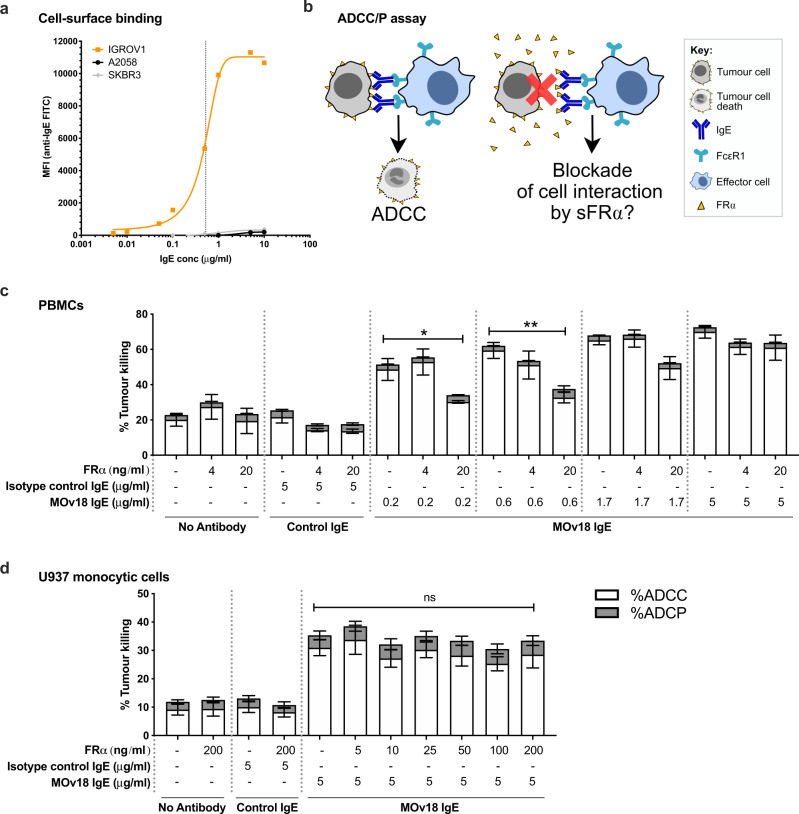
Potential blockade of efficacy of a FRα-targeted therapeutic antibody candidate by soluble FRα. **a** Binding of MOv18 IgE to FRα-expressing IGROV1 ovarian, but not to melanoma (A2058) or breast (SKBR3), cancer cells (EC50 = 0.53 µg/ml for binding to IGROV1 cells, indicated by vertical dotted line). **b** Schematic of potential blockade of MOv18 IgE anti-tumour function by sFRα. **c** Using PBMCs, the level of antibody-dependent cellular cytotoxicity (ADCC) mediated by the FRα-specific antibody MOv18 IgE was significantly reduced by a high concentration of FRα antigen (20 ng/ml) where MOv18 IgE was introduced at non-saturating concentrations of cancer cell-associated FRα (0.2 and 0.6 µg/ml; below or at the EC50 as shown in **a**). High concentrations of FRα antigen (20 ng/ml) did not block ADCC mediated by concentrations of MOv18 IgE above the EC50 (see **a**). **d** Similarly, with U937 monocytic cells, ADCC mediated by a saturating concentration of MOv18 IgE (5 µg/ml; see **a**) was not blocked by supraphysiological concentrations of FRα antigen (200 ng/ml). Antibody-dependent cellular phagocytosis (ADCP) was not mediated by MOv18 IgE. *N* = 6 and 4 independent experiments, respectively. *P ≤ 0.05, **P ≤ 0.01, ns not significantly different. Statistical test: one-way ANOVA with Kruskal–Wallis multiple comparisons. Error bars represent the standard error of mean (SEM).

We next evaluated whether the efficacy of anti-FRα treatments may be inhibited by the presence of free FRα in the circulation (Fig. 6). The FRα-specific IgE therapeutic candidate, MOv18 IgE [41, 44–47], bound to FRα-expressing IGROV1 ovarian cancer cells in a concentration dependent manner (EC50 = 0.53 µg/ml), but not to non-FRα-expressing A2058 and SKBR3 melanoma and breast cancer cells (Fig. 6a). We then explored the impact of circulating FRα on anti-FRα IgE antibody-dependent cellular cytotoxicity (ADCC) of cancer cells (Fig. 6b). ADCC of FRα-expressing IGROV1 cancer cells by human peripheral blood mononuclear cells (PBMCs) mediated by low concentrations of MOv18 IgE below or at the EC50 (0.2 or 0.6 μg/ml), were significantly-reduced by high (20 ng/ml), but not by low (4 ng/ml), recombinant FRα concentrations (≥20 ng/ml being the serum concentration measured in the top 8% of our patients) (Fig. 6c). In contrast, ADCC mediated by MOv18 IgE at levels close to target saturation (1.7 μg/ml) was somewhat, but not significantly perturbed. Furthermore, at saturating levels of IgE (5 μg/ml), ADCC by both PBMCs (Fig. 5c) and U937 human monocytic cells (Fig. 6d) was not reduced by the presence of even the highest levels of soluble FRα (20 ng/ml and 200 ng/ml, respectively).

These data suggest that the efficacy of anti-FRα antibodies may be impaired by high concentrations of circulating free FRα antigen (as measured in the 8% of ovarian cancer patients with the highest levels of sFRα; see Fig. 4a). However these partial blocking effects of free FRα were overcome by higher doses of the therapeutic candidate.

## Discussion

It has been previously reported that FRα may be a potential therapeutic target for ovarian cancer, and that this tumour-associated antigen is also shed from the tumour into the patient circulation [14, 25, 48, 49]. However, extensive studies to identify the levels of this marker in relation to disease burden and treatment have been lacking. In this study we evaluated FRα tumour expression and levels of sFRα in the circulation, with the aim of further characterising the clinico-pathologic correlates of this potential clinical biomarker, and of exploring the role of sFRα as a surrogate for tumoural FRα expression, tumour burden and response to treatment.

We first confirmed that gene expression of *FOLR1* (for FRα) and of members of the folate signalling pathway (FOLH1, TYMS and DHFR), were elevated in ovarian cancer cell lines and ovarian cancer tissues, compared to normal tissues (Fig. 2 and Supplementary Fig. 1). We furthermore evaluated FRα in normal tissues (*N* = 142), ovarian tumours (*N* = 316), patient serum (*N* = 143), and serum from healthy volunteers (*N* = 61) (Fig. 1). While no membrane or cytoplasmic FRα staining was observed in a broad range of normal tissues evaluated by IHC (Fig. 2), around half of ovarian tumour samples expressed FRα, either in the cytoplasm, on the cell membrane, or both, with 60% of the high-grade serous tumours being positive for FRα expression (Figs. 2 and 3). These data are consistent with previously published studies reporting FRα expression by 75-85% of high-grade serous samples [21, 22, 50]. As previously observed [14, 25], sFRα levels were significantly raised in patients, compared to healthy subjects, supporting the potential of sFRα as a serum marker for disease (Fig. 4). To explore the clinical correlates of FRα, we considered the levels of sFRα in different patient cohorts. The concentration of sFRα and the proportion of patients with detectable sFRα were greater in patients at the start of neo-adjuvant or palliative treatments, compared to patients commencing adjuvant chemotherapy, in whom debulking surgery had recently been performed.

Since we measured higher levels of sFRα in ovarian cancer patient cohorts likely to have a higher disease burden (namely those commencing neo-adjuvant and palliative chemotherapy), we developed a scoring system to quantify the burden of disease as observed in concurrent imaging (Fig. 5). As expected, tumour burden scores were significantly higher at baseline timepoints for patients in the neo-adjuvant and palliative cohorts, compared to the adjuvant cohort. In addition, in the neo-adjuvant and palliative cohorts, the tumour burden score showed a downward trend across longitudinal treatment timepoints. Circulating sFRα levels were also observed to fall across sequential treatment timepoints, however beta coefficients demonstrated that the changes were more marked between early treatment timepoints, compared to a more delayed decline in tumour burden score. Furthermore, ROC curve analysis showed that sFRα levels were found to be associated with tumour burden score (Fig. 5e). Taken together, our findings suggest that sFRα should be further explored as a useful non-invasive marker, in evaluating disease burden and measuring response to treatment in some patients, as well as selection of patients for potential FRα-targeting therapies. Since our analyses were performed on a limited number of patient samples, future studies in larger data sets are required.

In addition to exploring the clinical correlates of sFRα, we examined the possible role of sFRα as a surrogate for tumour FRα expression. Levels of sFRα were significantly higher in patients with tumours expressing FRα on the cell membrane, compared to patients with tumours negative for FRα. Detectable sFRα in the patient circulation was predictive of FRα-positive tumour cell membrane expression (Fig. 4a). These data are in concordance with the hypothesis that tumours expressing FRα can shed this antigen into the circulation [25, 48].

A number of FRα-targeting therapeutics are currently in pre-clinical and clinical development [12, 26, 31], including our own FRα-specific IgE antibody (MOv18 IgE) [41, 44–47, 51]. Here we demonstrate that the patients with FRα-expressing tumours, which could be targeted with such therapies, are likely to have circulating sFRα. Therefore, we evaluated whether this soluble tumour-associated antigen could act as a decoy to block cancer cell recognition and consequently treatment efficacy (Fig. 6). High concentrations of FRα, equivalent to those measured in sera from 8% of patients, perturbed the ADCC of FRα-expressing tumour cells by immune effector cells mediated by low concentrations of MOv18 IgE. However, at higher antibody concentrations, the anti-tumoural potency of MOv18 IgE was unaltered, suggesting that the number of antigen-binding sites on the antibody outweighed any blocking effects of free FRα antigen and were thus sufficient to trigger cancer cell cytotoxicity. To overcome any decoy functions of sFRα, in future it may be necessary to increase the required therapeutic dose of anti-FRα therapies in the minority of patients with the greatest levels of sFRα. In the case of MOv18 IgE, this limitation may be overcome by the kinetic properties of this antibody class: once engaged with FcεRI-expressing immune effector cells, MOv18 IgE may be trafficked away from the circulation into the tumour where the majority of FRα may be present on the target tumour cells rather than in soluble form. Predictions for the safe administration of FRα-specific IgE antibody in patients with detectable sFRα (and/or anti-FRα autoantibodies) in the circulation have been previously made using ex vivo mast cell degranulation and basophil activation test (BAT) approaches [37, 39]. The associations between MOv18 IgE efficacy and safety, with levels of sFRα, will be studied in the ongoing Phase I clinical trial of this therapeutic candidate (ClinicalTrials.gov Identifier NCT02546921).

## Conclusions

We demonstrate that FRα overexpression, at the cell membrane and in the cytoplasm, is associated with ovarian cancer, presenting a promising therapeutic target. sFRα shed into the circulation of ovarian cancer patients was most markedly observed at the start of neo-adjuvant and palliative treatment and was predictive of tumour cell surface but not of cytoplasmic FRα expression. Furthermore, sFRα was reflective of patients’ disease burden and response to standard treatment, as measured with our devised tumour burden score. Therefore, sFRα may present a useful and dynamic non-invasive marker for tumour FRα expression, which could be used to monitor patient response to treatment, and which may reflect potential for resistance to FRα-targeting therapies or a requirement for increased drug administration if detected at high levels. The potential utility of sFRα should be evaluated further in prospective studies of large patient cohorts.

## Supplementary information

Supplementary Material

Bax et al. Reporting Summary Checklist

## Data Availability

The data that support the findings of this study are available from the corresponding author upon reasonable request.
