# Supplementary material for: Folate receptor alpha in ovarian cancer tissue and patient serum is associated with disease burden and treatment outcomes
Source: Br J Cancer. 2022 Nov 19;128(2):342–53. doi: 10.1038/s41416-022-02031-x (PMC9902484; doi:10.1038/s41416-022-02031-x)
Supplement: Supplementary file 1 — Supplementary Material [file 41416_2022_2031_MOESM1_ESM.pdf]

Supplementary Table 1: Characteristics of ovarian patients that provided serum samples.

	**Neo-adjuvant**	**Adjuvant**	**Palliative**
**Number of patients**	57	33	53
**Age** – median (range)	65 (25-86)	59 (28-79)	64 (43-87)
**Tumour histology** – total number (%)			
Epithelial ovarian			
High grade serous	54 (94.7)	18 (54.5)	44 (83.0)
Low grade serous	1 (1.8)	2 (6.1)	1 (1.9)
Endometrioid	1 (1.8)	1 (3.0)	1 (1.9)
Clear cell	0 (0)	9 (27.3)	3 (5.7)
Mixed	0 (0)	1 (3.0)	1 (1.9)
Adult granulosa cell	0 (0)	0 (0)	1 (1.9)
High grade serous endometrial	1 (1.8)	0 (0)	0 (0)
Carcinosarcoma	0 (0)	2 (6.1)	2 (3.8)
**FIGO-stage** – total number (%)			
Stage I	0 (0)	10 (30.3)	8 (15.1)
Stage II	0 (0)	5 (15.2)	1 (1.9)
Stage III	31 (54.4)	17 (51.5)	37 (69.8)
Stage IV	26 (45.6)	1 (3.0)	7 (13.2)
**Debulking surgery** – total number (%)			
Optimal	29 (50.9)	30 (90.9)	N/A
Suboptimal	21 (36.8)	2 (6.1)	N/A
No debulking surgery	7 (12.3)	1 (3.0)	N/A
**Primary tumour** – total number (%)			
Ovary	45 (78.9)	24 (72.7)	42 (79.2)
Fallopian tube	8 (14.0)	9 (27.3)	7 (13.2)
Primary peritoneal	3 (5.3)	0 (0)	4 (7.5)
Endometrium	1 (1.8)	0 (0)	0 (0)
**Menopausal status** – total number (%)			
Post-menopausal	45 (78.9)	22 (66.7)	45 (84.9)
Pre-menopausal	2 (3.5)	2 (6.1)	0 (0)
Unknown	10 (17.5)	9 (27.3)	8 (15.1)

Supplementary Table 2: Tumour Burden Score

	Example 1	Example 2	Example 3
**Site of disease (1 point per site)**			
Lung	0	0	0
Pleural	0	0	0
Pleural Effusion	0	1	0
Liver	0	1	1
Spleen	0	0	1
Soft Tissue	0	0	0
Peritoneal Nodularity	0	1	1
Pelvic Mass	0	1	1
Ascites	1	1	1
Lymph nodes	1	1	4
Bone	0	0	0
**Sub-Total**	**2**	**6**	**9**
**Number of metastases per site (1-2 = 1 point), (2-4 = 2 points), (≥5 = 3 points)**			
Lung	0	0	0
Pleural	0	0	0
Pleural Effusion	0	1	0
Liver	0	1	2
Spleen	0	0	2
Soft Tissue	0	0	0
Peritoneal Nodularity	0	1	1
Pelvic Mass	0	1	1
Ascites	1	1	1
Lymph nodes	1	1	1
Bone	0	0	0
**Sub-Total**	**2**	**6**	**8**
**Maximum metastasis diameter per site (<2cm = 1 point), (2-5cm = 2 points), (>5cm = 3 points)**			
Lung	0	0	0
Pleural	0	0	0
Pleural Effusion	0	1	0
Liver	0	3	3
Spleen	0	0	3
Soft Tissue	0	0	0
Peritoneal Nodularity	0	1	2
Pelvic Mass	0	3	3
Ascites	1	1	1
Lymph nodes	1	1	2
Bone	0	0	0
**Sub-Total**	**2**	**10**	**14**
**Total Tumour Burden Score**	**(2+2+2) = 6**	**(6+6+10) = 22**	**(9+8+14) = 31**

Supp. Figure 1

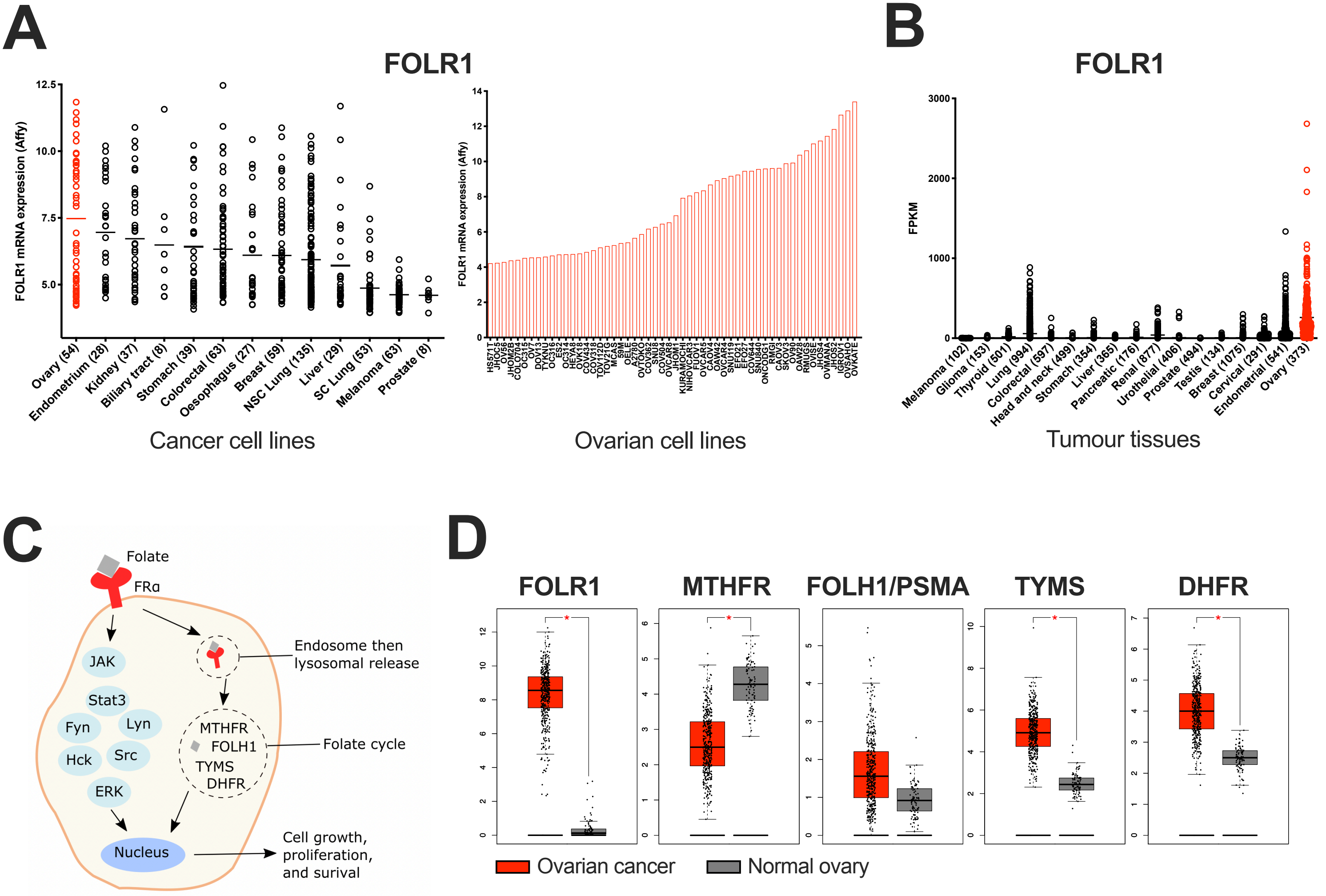

Supp. Figure 2

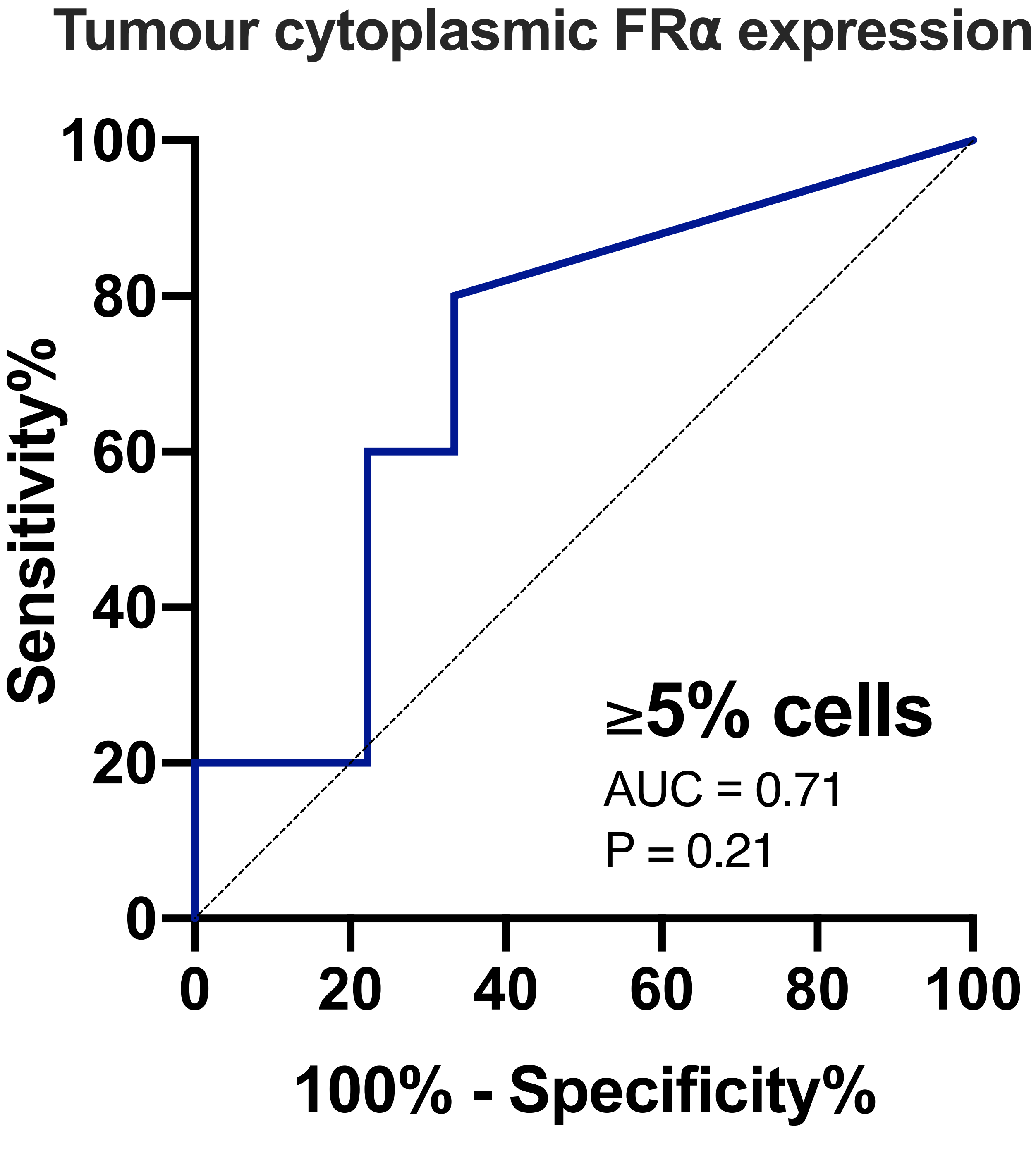

Supp. Figure 3

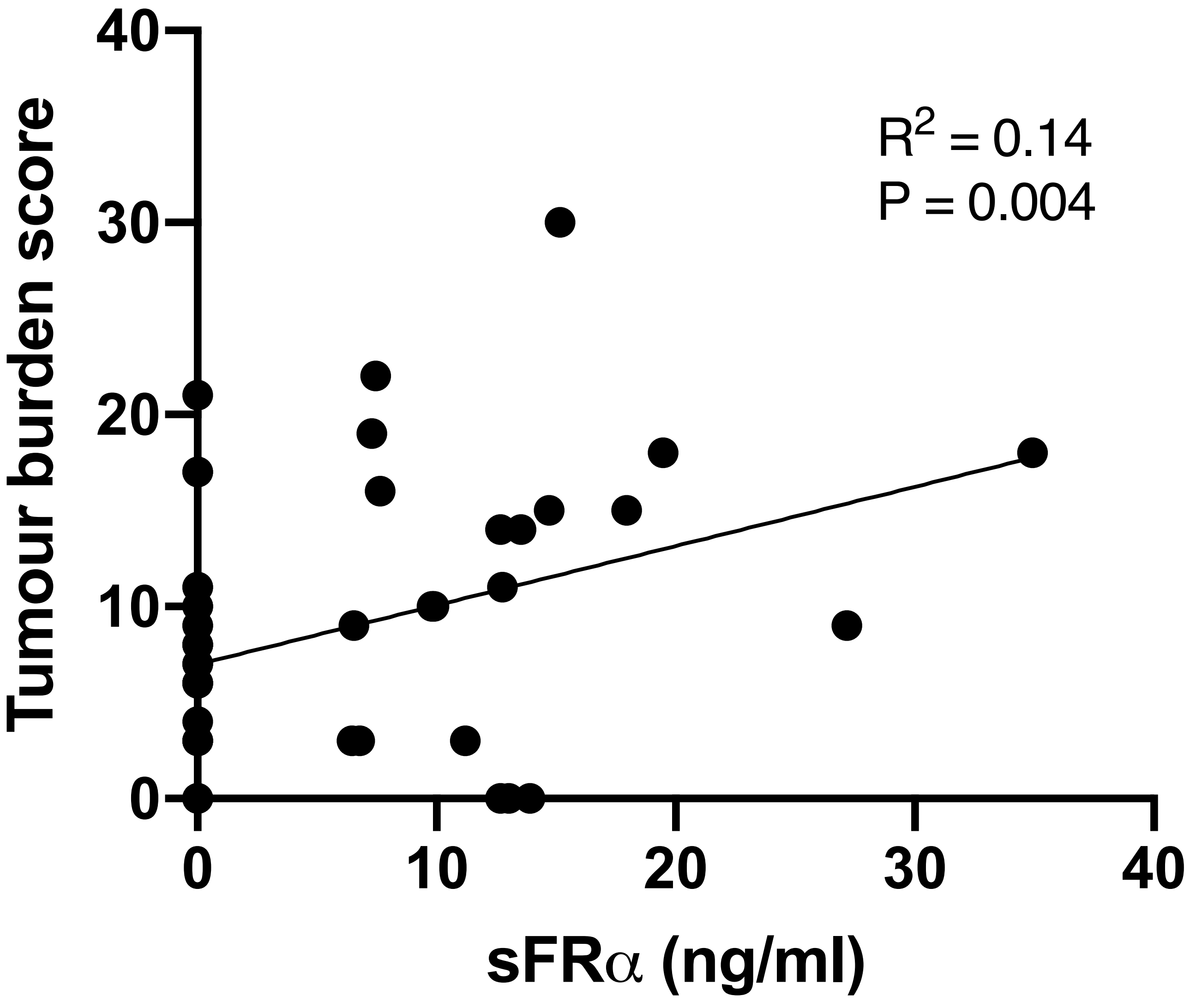

Supplementary Figure Legends

Supplementary Figure 1: High *FOLR1* gene expression in ovarian cancers. A: Gene expression levels of *FOLR1* (encoding FRα) measured in human cancer cell lines (left, 10 top- and 3-lowest expressing cancer types shown, N numbers indicated in parentheses) and across ovarian cancer cell lines (right) (Cancer Cell Line Encyclopedia (CCLE) online tool) indicate high relative expression in ovarian cancer cells. B: Gene expression levels (fragments per kilobase of exon per million mapped fragments, FPKM) were also elevated in human ovarian cancer tissues (N numbers indicated in parentheses; The Human Protein Atlas online tool [1,2]). C: The folate/FRα signaling pathway involves intracellular molecules, e.g., *MTHFR*, *FOLH1*, *TYMS*, and *DHFR*, and is associated with cell growth, proliferation and survival. D: *FOLR1*, and folate pathway signaling molecules are differentially expressed in ovarian cancer in relation to normal ovary tissues (Gene Expression Profiling Interactive Analysis (GEPIA) online tool [3]; ovarian tumours, N=426; normal ovary tissues, N=88).

Supplementary Figure 2: Receiver operating characteristic (ROC) curve analysis evaluating the capability of sFRα to predict cytoplasmic FRα expression in tumours. In the neo-adjuvant treatment-naïve patient group, sFRα concentration was not predictive of cytoplasmic FRα expression in the patients' tumour. Statistical test: receiver operating characteristic (ROC) curve analyses.

Supplementary Figure 3: Correlation between tumour burden score and sFRα. In the neo-adjuvant treatment-naïve patient group, a modest correlation was observed between tumour burden score and the level of circulating of sFRα.

1 Gyorffy, B., Lanczky, A. & Szallasi, Z. Implementing an online tool for genome-wide validation of survival-associated biomarkers in ovarian-cancer using microarray data from 1287 patients. *Endocr Relat Cancer* **19**, 197-208 (2012).

2 Uhlen, M., Zhang, C., Lee, S., Sjostedt, E., Fagerberg, L., Bidkhori, G. *et al.* A pathology atlas of the human cancer transcriptome. *Science* **357** (2017).

3 Tang, Z., Li, C., Kang, B., Gao, G., Li, C. & Zhang, Z. GEPIA: a web server for cancer and normal gene expression profiling and interactive analyses. *Nucleic Acids Res* **45**, W98-W102 (2017).
